# L’hépatite virale B en milieu de soins: facteurs associés à la vaccination chez les élèves professionnels de santé de l’Ecole nationale de santé publique de Ouagadougou, Burkina Faso

**DOI:** 10.11604/pamj.2022.42.227.30672

**Published:** 2022-07-22

**Authors:** Pengdewendé Maurice Sawadogo, Larba Lankoandé, Désiré Lucien Dahourou, Koiné Maxime Drabo

**Affiliations:** 1 Institut Supérieure des Sciences de la Population de l’Université Joseph Ki-Zerbo, Ouagadougou, Burkina Faso,; 2 Observatoire National de la santé de la population, Ouagadougou, Burkina Faso,; 3 Centre National de la Recherche Scientifique et Technologique (CNRST), Ouagadougou, Burkina Faso,; 4 Direction de la Formation Supérieure en Sciences de la Santé (DFSSS), Ouagadougou, Burkina Faso

**Keywords:** Connaissances, élèves, professionnels de santé, vaccination, hépatite B, Knowledge, health professional students, vaccination, hepatitis B

## Abstract

**Introduction:**

l´hépatite B est une maladie grave évitable par la vaccination. Pourtant, peu de professionnels de santé sont vaccinés contre cette maladie au Burkina Faso. Cet article examine les facteurs associés à la vaccination chez des élèves professionnels de santé.

**Méthodes:**

l´étude a été conduite auprès des élèves professionnels de santé de l´école nationale de santé publique de Ouagadougou au Burkina Faso. La collecte de données s´est déroulée du 26 mai au 1^er^ juin 2020. Les participants aléatoirement choisis ont été soumis à un questionnaire auto-administré. Les facteurs associés à la vaccination ont été déterminés à l´aide d´une régression logistique multivariable et les résultats ont été présentés sous forme de odds ratio ajustés avec des intervalles de confiance à 95%.

**Résultats:**

au total, 410 élèves professionnels de santé ont participé à l´étude. Ils étaient majoritairement de sexe féminin soit 68,8% (293/410) et leur âge moyen était de 25,71 ans (± 3,57). Parmi ces élèves, 31% (127/410) était complètement vaccinés contre l´hépatite B. Dans le modèle multivariable, la connaissance du mode de transmission, des complications de la maladie, et des risques d´expositions en milieu de soins étaient associées à la vaccination contre l´hépatite B.

**Conclusion:**

la faible couverture vaccinale des élèves professionnels de santé est liée à une insuffisance de connaissances sur l´hépatite B. Le renforcement des connaissances de ce groupe à risque sur cette maladie s´avère nécessaire.

## Abstract (en)

**Introduction:**

hepatitis B is a severe disease that can be prevented by vaccination. However, few healthcare professionals, a group at high risk of contamination, are vaccinated against this disease in Burkina Faso. We conducted a study of healthcare professional students to evaluate their knowledge and factors associated with their propensity toward Hepatitis B vaccine.

**Methods:**

we conducted a cross-sectional, descriptive and explanatory study of 410 healthcare professional students of the National School of Public Health of Ouagadougou, Burkina Faso. Data were collected from 26 to 1 June 2020. Participants were randomly selected and were given a self-administered questionnaire.

**Results:**

less than one third of healthcare professional students were fully vaccinated against hepatitis B. A small proportion of healthcare professional students were aware of the three modes of transmission (46.6%), risks of exposure in the healthcare setting (31.2%) and disease complications (26.6%). Multivariate logistic regression showed that healthcare professional students´ awareness of the risks of exposure in the healthcare setting and complications of the disease were statistically associated with hepatitis B vaccination.

**Conclusion:**

strengthening the knowledge of healthcare professional students is necessary to improve vaccination coverage in this risk group.

## Introduction

L´hépatite B est une maladie grave causée par le virus de l´hépatite B (VHB). La maladie peut être foudroyante et causer le décès dans un tableau de cancer primitif du foie. Elle affecte 3,5% de la population mondiale et cause une infection chronique chez 257 millions de personnes [1]. Au Burkina Faso, elle touchait 14,5% de la population générale en 2014 [2].

Le VHB est l´un des 20 plus graves agents pathogènes à diffusion hématogène qui menacent la santé des travailleurs [3,4]. Chaque année, plus de 66 000 professionnels de santé sont infectés par ce virus en raison de leur occupation [5]. Chez ces professionnels, le VHB se transmet principalement à l´occasion des blessures par piqûres d´aiguilles et d´objets tranchants au cours de l´administration des soins. Ainsi, les infirmiers/ères, les sages-femmes et les techniciens médicaux qui administrent les soins ou manipulent les échantillons de liquides biologiques sont particulièrement exposés au risque de contamination [6]. Les élèves professionnels de santé qui posent également ces actes sont aussi exposés. Chez ces derniers, le risque est d´autant plus élevé du fait de leur manque d´expériences dans l´administration des soins ou dans la manipulation des échantillons biologiques [6]. Au Burkina Faso, l´ampleur de l´infection à VHB chez les professionnels de santé n´est pas connue. Toutefois, son ampleur pourrait être important au regard de la récurrence des expositions accidentelles au sang ou aux liquides biologiques rapportées chez le personnel soignant [5,7-9].

Au regard du risque encouru, l´Organisation mondiale de la santé (OMS) a recommandé la vaccination obligatoire contre l´hépatite B pour les personnes exerçant la profession de soignant [10]. Aussi, le Code de Santé Publique du Burkina Faso exige la vaccination pour toute personne dont l´activité professionnelle expose à des risques de contamination [11]. Toutefois, en 2010, seulement 47,7% des professionnels de santé du Burkina Faso était vacciné contre le VHB [12]. Aussi, peu d´études ont analysé les facteurs explicatifs de la vaccination chez les soignants dans le contexte du Burkina Faso.

Cette étude examine les facteurs associés à la vaccination contre l´hépatite B en milieu de soins au Burkina Faso. Elle s´est focalisée sur les élèves professionnels de santé qui est un sous-groupe de soignants particulièrement exposée au risque de contamination.

## Méthodes

**Conception et cadre de l´étude:** cette étude est de type transversal, descriptive et analytique. Elle a été conduite dans la direction régionale de l´École Nationale de Santé Publique (ENSP) de Ouagadougou au Burkina Faso. L´ENSP est une institution de formation des professionnels de santé non-médecins notamment des infirmiers, des sages-femmes et des technologistes biomédicaux. Les élèves sont admis dans cette école par concours direct ou professionnel ou encore par inscription à titre privé. La formation de ces prestataires de soins comprend une phase théorique au cours de laquelle, les maladies les plus fréquentes (y compris l´hépatite B) et les techniques de soins sont enseignées en salle. Après cette phase théorique, les élèves rejoignent les centres de santé où ils mettent en pratique les enseignements reçus, notamment l´administration des soins aux malades, les prélèvements de sang et d´autres liquides biologiques, l´analyse des échantillons.

**Population d´étude:** elle est constituée des élèves infirmiers, sages-femmes ou technologistes biomédicaux. Nous avons inclus des élèves de première année d´étude, de deuxième année ou de troisième année (année terminale) qui ont consenti participer à l´étude. Aucun critère d´exclusion n´a été défini. La formule de Schwartz a été utilisée pour le calcul de la taille de l´échantillon. La proportion d´élèves professionnels de santé vaccinés contre le VHB étant inconnue dans notre contexte, nous l´avons estimé à 50%. Avec une précision de 5% et un taux de non-réponse de 10%, la taille de l´échantillon était de 422 élèves. Cet échantillon a été réparti entre infirmiers (39,8 %), sages-femmes (43,4%) et technologistes biomédicaux (16,8%) proportionnellement à leur effectif dans l´école. La sélection des élèves a été faite par un tirage aléatoire systématique en utilisant la liste (par ordre alphabétique) des élèves de chaque filière.

**Collecte des données:** elle s´est tenue du 26 mai au 1^er^ juin 2020 et a été effectuée par des agents formés à cet effet. La collecte s´est faite au moyen d´un questionnaire qui a préalablement été testé auprès des élèves d´une école privée de santé dénommée Sainte Edwige qui est aussi située dans la ville de Ouagadougou.

**Variables d´étude:** la variable dépendante était le statut vaccinal contre l´hépatite virale B et a été obtenu à travers la déclaration de l´enquêtée. Pour les besoins des analyses, la variable dépendante prend la valeur 1 quand l´élève déclare avoir reçu au moins une dose de vaccin contre l´hépatite B et la valeur 0 dans le cas contraire. Les variables indépendantes étaient constituées des caractéristiques sociodémographiques (âge, sexe, mode d´admission, la réligion et le niveau d´instruction du parent le plus proche de l´élève professionnel) et les connaissances sur la maladie (mode de transmission, risques d´exposition en milieu professionnel, complications et le niveau de formation). La distribution de fréquence des variables à l´étude sont présentés dans le Tableau 1.

**Tableau 1:** répartition des élèves professionnels selon les caractéristiques à l´étude, Burkina Faso, 2020 (n=410)

Variables	Effectifs	Proportions (%)
**Groupe d´âge**		
Moins de 20 ans	30	7,30
20-29 ans	315	76,80
30 ans et +	65	15,90
**Sexe du répondant**		
Féminin	282	68,80
Masculin	128	31,20
Mode d´admission à l´ENSP		
Concours	113	27,60
Autres modes d´admission	297	72,40
**Niveau d'instruction du parent le plus proche de l´élève professionnel**		
Aucun	73	17,80
Primaire	81	19,80
Secondaire et plus	256	62,40
**Niveau de connaissance sur les modes de transmissions**		
Aucun	42	10,20
Un ou deux modes	177	43,20
Trois modes	191	46,60
**Niveau de connaissance des risques d´expositions en milieu de soins**		
Aucun	133	32,40
Moyen	149	36,40
Bon	128	31,20
**Niveau de connaissance sur des signes de complications**		
Aucun	149	39,30
Un signe	107	34,10
Deux signes	154	26,60
**Niveau de formation à l´ENSP**		
1^e^ Année	95	32,20
2^e^ Année	156	38,00
3^e^ Année	159	38,80
**Total**	**410**	**100**

**Analyses statistiques:** l´analyse statistique, dans un premier temps, a consisté à décrire les variables à l´aide de fréquences et de pourcentages (%). Elle a été suivie d´une analyse uni variable pour étudier l´association entre chaque covariable et l´état vaccinal. Les variables dont la p-value est ≤ 20% à l´analyse uni variable ont été sélectionnées pour faire partie de l´analyse multi-variable. Les analyses multivariables ont été effectuées à travers la régression logistique binaire. Un seuil de significativité de 5% a été fixé pour l´ensemble des estimations faites dans le cadre de la présente étude. Les données collectées ont été saisies à l´aide du logiciel épi info 7.2. Les analyses statistiques ont été effectuées avec le logiciel *Statistical Package for the Social Sciences (SPSS)* version 25.0.

**Considérations éthiques:** l´étude a requis l´autorisation du directeur général de l´ENSP (n°323 MS/SG/ENSP/DRO du 20/03/2020). La participation des élèves était volontaire. Un consentement écrit libre et éclairé a été obtenu avant l´administration du questionnaire.

## Résultats

Sur un échantillon prévisionnel de 422 élèves professionnels, 410 avaient pris part à l´étude, soit un taux de participation de 97,15% (410/422). L´âge moyen des élèves était de 25,71 ans (±3,57). Les élèves âgés de 20-29 ans représentaient 76,8% (65/410). Les élèves de sexe féminin constituaient 68,8% (282/410) de l´échantillon. Aussi, 72,40% (297/410) des élèves était inscrit à titre privé et 62,40 % (256/410) des élèves avait un parent proche ayant un niveau secondaire et plus. En matière de connaissances sur l´hépatite B, il est à noter que 46,60% (191/410) avait pu citer les trois modes de transmission, 31,20 % (128/410) avait une bonne connaissance des risques d´expositions en milieu professionnel et 26,60 % (154/410) connaissait au moins deux (02) signes de complications de la maladie (Tableau 1).

L´analyse a montré que 31% (127/410) des élèves professionnels avait reçu au moins une dose du vaccin anti-VHB. À l´analyse univariable, l´âge (p=0,002), le sexe (p=0,015), le mode d´admission à l´ENSP (p=0,031) et le niveau d´instruction du parent le plus proche de l´élève professionnel (p=0,002) étaient associés au statut vaccinal au seuil de 10%. De même, la connaissance du mode de transmission du VHB (p=0,001), des risques d´exposition en milieu de soins (p=0,002), des complications de la maladie (p=0,000) et le niveau de formation à l´ENSP (p=0,000) étaient associés au statut vaccinal au seuil de 10% (Tableau 2). Toutes les co-variables dont les odds ratio brutes étaient significatifs au seuil de 10% ont été inclus dans l´analyse multivariable.

**Tableau 2:** relation entre le statut vaccinal des élèves, leurs caractéristiques sociodémographiques et leurs connaissances sur le VHB, Burkina Faso, 2020 (n=410)

Variables	Effectif	Statut vaccinal		P-value
		Non vacciné (%)	Vacciné (%)	
**Caractéristiques sociodémographiques**				
**Groupe d´âge**				
Moins de 20 ans	25	92,00	8,00	0,002
20-29 ans	312	70,30	29,70	
30 ans et plus	72	55,60	44,40	
**Sexe**				
Masculin	117	77,80	22,20	0,015
Féminin	293	65,50	34,50	
**Mode d´admission à l´ENSP**				
Par concours	113	77,00	23,00	0,031
Inscription à titre privée	297	66,00	34,00	
**Niveau d'instruction du parent le plus proche de l´élève professionnel**				
Aucun	61	85,20	14,80	0,002
Primaire	74	75,70	24,30	
Secondaire et plus	275	63,60	36,40	
**Réligion de l´enquêté**				
Catholiques	113	66,5	33,5	0,119
Protestants	56	63,6	36,4	
Musulmans	114	75,0	25,0	
**Connaissance de la maladie**				
**Modes de transmission**				
Aucun	42	92,90	7,10	0,001
1-2 modes	177	70,10	29,90	
3-modes	191	62,80	37,20	
**Risques d´expositions en milieu de soins**				
Aucun	133	79,70	20,30	0,002
Moyen	149	67,10	32,90	
Élevé	128	60,20	39,80	
**Complications de la maladie**				
Aucun	161	82,00	18,00	0,000
1 complication	140	70,70	29,30	
2 complications et +	109	47,70	52,30	
**Niveau de formation à l´ENSP**				
1^e^ année	95	87,40	12,60	0,000
2^e^ année	156	69,90	30,10	
3^e^ année	159	57,20	42,80	
Total	410	31,0	69,0	

À l´analyse multivariable, il est ressorti des caractéristiques sociodémographiques et des variables de connaissances qui étaient statistiquement associées à la propension à se faire vacciner contre l´hépatite B. Au titre des caractéristiques sociodémographiques, l´on note qu´être de sexe féminin (ORa: 0,55 IC 95% 0,31-0,98; p=0.044) était associé à une faible probabilité de se faire vacciner contre l´hépatite B. Par contre, les élèves inscrits à titre privé (ORa: 2, 06 IC95% 1,14-3,71; p=0,016) ou ceux dont le parent le plus proche a un niveau d´instruction secondaire et plus (ORa: 2,34 IC95% 1,03-5,35; p=0.042) étaient plus susceptibles d´être vaccinés contre le VHB. Aussi, le fait de connaitre une à deux modes de transmission (ORa: 4,36 IC 95% 1,16-16,41; p=0,029) ou encore trois modes de transmission du VHB (ORa: 4,87 IC 1,28-18,47; p=0,020) était associé à une forte propension à se faire vacciner. Aussi, la connaissance des risques d´exposition en milieu de soins (ORa: 1,93 IC 95% 1,03-3,60 p=0,039) et la connaissance d´au moins deux signes de complications de la maladie (ORa: 3,01 IC 95% 1,61-5,61 p=0,001) étaient associées à une forte propension à se faire vacciner contre l´hépatite B. Enfin, les élèves en troisième année d´étude (ORa: 2,91 IC 95% 1,02-5,14 p=0,044) étaient plus susceptibles d´être vaccinés contre l´hépatite comparativement aux élèves en première année (Tableau 3).

**Tableau 3:** effets bruts et effets ajustés des caractéristiques sociodémographiques et des connaissances des élèves sur la vaccination contre l´hépatite B, 2020 (n=410)

Variables	Vaccination contre le VHB			
	OR brute [IC 95%]	P-value	OR ajusté [IC 95%]	P-value
**Caractéristiques sociodémographiques**				
**Groupe d´âge (réf: moins de 20 ans)**				
20-29 ans	4,86 [1,12; 21,04]	0 ,034	2,63 [0,56; 12,23]	0,182
30 ans et plus	9,20 [2,017; 41,97]	0,004	4,54 [0,88; 23,29]	0,060
**Sexe (réf: masculin)**				
Féminin	0,54 [0,33; 0,89]	0.016	0,55 [0,31; 0,98]	0.044
**Mode d´admission (référence: par concours)**				
Inscription privée	1,72 [1,04; 2,84]	0.033	2, 06 [1,14; 3,71]	0,016
**Niveau d´instruction du parent le plus proche de l´élève professionnel (réf: aucun)**				
Primaire	1,86 [0,76; 4,49]	0,17	1,30 [0,49; 3,45]	0,592
Secondaire et plus	3,30 [1,46; 6,98]	0,002	2,34 [1,03; 5,35]	0.042
**Connaissances des enquêtés sur l´hépatite b**				
**Modes de transmission (réf: aucun)**				
1-2 modes	5,56 [1,64; 18,77]	0,006	4,36 [1,16; 16,41]	0,029
3 modes	7,69 [2,29; 25,81]	0,001	4,87 [1,28; 18,47]	0,020
**Risques d´expositions en milieu de soins (réf: aucun)**				
Moyen	1,92 [1,12; 3,31]	0,017	1,93 [1,03; 3,60]	0,039
Élevé	2,60 [1,49; 4,51]	0,001	1,77 [1,01; 3,27]	0,060
**Complications de la maladie (réf: aucun)**				
1 signe de complications	1,89 [1,09; 3,24]	0,022	1,23 [0,67; 2,24]	0,498
2 signes de complications et plus	4,99 [2,89; 8,65]	0,000	3,01 [1,61; 5,61]	0,001
**Niveau de formation à l´ENSP (réf: 1e année)**				
2^e^ année	2,98 [1,48; 5,97]	0,002	1,99 [0,79; 4,06]	0,159
3^e^ année	5,17 [2,61; 10,22]	0,000	2,91 [1,02; 5,14]	0,044
**Constante**			0,003	
**Sig du modèle**				0,000
**R-deux de Nagelkerke**			0,265	

## Discussion

Cette étude a examiné les facteurs associés à la vaccination contre l´hépatite B chez les élèves professionnels de santé. Il en ressort que des caractéristiques sociodémographiques des élèves et leurs connaissances sur la maladie étaient associés à leur propension à se faire vacciner. Les résultats de l´étude ont montré que seulement 31% des élèves était vacciné contre l´hépatite B. Ce niveau est inférieur à la couverture vaccinale chez les professionnels en activité puisque 47% d´entre eux était vacciné en 2010 [13]. Ainsi, environ deux élèves professionnels sur trois courent le risque d´être infecté par le VHB au cours de leur stage. Ces résultats appellent à des stratégies innovantes pour améliorer la couverture vaccinale des élèves professionnels de santé.

Il est aussi ressorti que les femmes sont moins susceptibles d´être vaccinées contre l´hépatite B, comparativement aux hommes. En Ouganda par contre les étudiants de sexe masculin étaient moins susceptibles d´être vaccinés, avec un ratio de prévalence de 0,79 (p < 1%) [14]. Cette plus grande propension des hommes à être vaccinés est due en partie au fait que la prévalence de l´hépatite B est plus élevé chez les hommes [2], ce qui fait qu´ils se sentent plus vulnérables que les femmes. Des études complémentaires s´avèrent nécessaires pour éclairer ces différences de comportement entre hommes et femmes dans le recours à la vaccination contre l´hépatite B au Burkina Faso.

En outre, les élèves professionnels qui se sont inscrits à titre privé sont deux fois plus susceptibles d´être vaccinés contre le VHB comparativement à ceux qui sont admis par concours. Des résultats similaires d´une plus forte propension à être vacciné chez les inscrits privés (comparativement aux étudiants pris en charge par le gouvernement) ont été rapportés en 2019 en Ouganda [14]. Ce résultat serait dû au fait que les élèves inscrits à titre privé auraient un niveau de revenu plus élevé leur permettant d´acheter le vaccin. Ainsi, une subvention des coûts du vaccin devrait permettre de booster la vaccination chez les élèves qui ont des moyens relativement limités.

Il est ressorti aussi une plus grande propension à la vaccination chez les élèves dont le proche-parent a un niveau d´instruction secondaire comparativement à leurs semblables de proche-parent ayant un niveau d´instruction moins élevé. Les parents ayant un niveau d´instruction élevé (secondaire et plus) occupent généralement des emplois bien rémunérés. Ainsi, ils peuvent acquérir les vaccins pour leurs enfants. Aussi, instruction rimant avec accès à une plus grande diversité de sources d´informations, ces parents instruits disposeraient de plus de connaissances sur l´hépatite B d´où une plus grande motivation à soutenir la vaccination de leurs enfants.

À côté des caractéristiques sociodémographiques, les connaissances sur l´hépatite virale B notamment les modes de transmission, les risques d´exposition, les complications de la maladie se sont avérées déterminantes pour le recours à la vaccination contre le VHB. Il est ressorti que seulement 46,60% avaient pu identifier les trois modes de transmission de la maladie. Des niveaux de connaissances de même ordre avaient été rapportés au Sénégal en 2017 [15]. En plus de la faible connaissance des modes de transmission, on note aussi que peu d´élèves (31,20%) connaissaient les risques d´exposition en milieu professionnel. Pourtant, dans le Nord-ouest de l´Éthiopie, plus de 80% des élèves professionnels avaient une connaissance jugée adéquate du VHB [16]. Comme il fallait s´y attendre, les élèves professionnels connaissant les modes de transmission ou ceux connaissant les risques d´exposition en milieu professionnel étaient plus susceptibles d´être vaccinés contre l´hépatite B comparativement à leurs collègues qui n´en connaissaient pas. Cette situation se justifierait par le fait que la connaissance des risques d´exposition et des modes de transmission suscite une prise de conscience des risques de contamination auxquels ils font face. Cet état de fait va amener l´élève-soignant à développer des stratégies de protection à travers entre autres la vaccination. Ce faible niveau de connaissances des modes de transmission et des risques d´exposition en milieu de soins est en partie lié à l´insuffisance de communication à leur endroit. Des programmes de communication sont donc utiles pour renforcer les connaissances des élèves en la matière.

Il est ressorti par ailleurs que seulement 60,70% avaient pu identifier au moins un signe de complications de l´hépatite B. Des résultats de même ordre avaient été mis en évidence en Inde à travers une étude qui a trouvé que la moitié des étudiants (50%) manquaient de connaissances sur les caractéristiques cliniques et les complications de l´hépatite B [17]. Les élèves professionnels connaissant les complications de l´hépatite sont plus susceptibles d´avoir été vacciné que ceux qui n´en connaissent pas. Cette propension à être vacciné augmente avec le niveau de connaissance des complications. À cet effet, les programmes de sensibilisation doivent viser l´amélioration des connaissances sur les complications de la maladie; ce qui favorisera l´adhésion des populations à la vaccination contre l´hépatite B.

Cette étude a contribué à combler une insuffisance de connaissances scientifiques sur l´hépatite B en milieu de soins au Burkina Faso. Elle a estimé pour la première fois la couverture vaccinale contre l´hépatite B chez les élèves professionnels de santé. Les résultats de cette étude qui a concerné des élèves de profil infirmier, sage-femme ou technologiste biomédical, peuvent être généralisés à l´ensemble des élèves professionnels de santé de même profil du Burkina Faso dans la mesure où c´est le même programme de formation qui leur est dispensé. Par contre, on pourrait avoir des résultats différents avec d´autres professionnels de santé, notamment les étudiants en médecine qui ne posent pas les mêmes actes et ne sont pas soumis au même programme de formation que ceux étudiés dans la présente étude. Une autre limite de cette étude est que l´état vaccinal a été recueilli sur déclaration de l´enquêté. La consultation de documents attestant du statut serait plus indiquée. Pour minimiser ce biais, nous avons insisté au cours de la formation des enquêteurs sur la nécessité de bien expliquer les objectifs de l´étude. Aussi, vu le niveau élevé d´instruction de la population étudiée, il est peu probable qu´ils confondent l´hépatite B avec d´autres maladies ou encore qu´ils déclarent un faux statut du fait de l´oubli. Ainsi, cette insuffisance aurait des effets marginaux sur les résultats de la présente étude.

**Financement:** les données utilisées pour la présente étude ont été collectées dans le cadre d´un mémoire de fin d´études d´un des auteurs. Pour la rédaction de leur mémoire de fin d´études, l´institution de formation a alloué une somme forfaitaire (environ 80 dollars USD) à chaque étudiant pour soutenir la collecte des données et la reprographie des documents. Ce financement qui ne couvre qu´une infime partie du coût estimatif de cette étude n´a altéré aucunement son intégrité.

## Conclusion

La présente étude a permis de connaître que seulement 31% des élèves professionnels de la direction régionale de l´ENSP de Ouagadougou étaient vaccinés contre le VHB en 2020. Elle a mis en exergue qu´une insuffisance de connaissances sur la maladie et des barrières financières expliquaient le faible recours à la vaccination parmi les élèves professionnels de santé au Burkina Faso. Ainsi, la direction des écoles professionnelles de santé doit mettre à la disposition des élèves des mécanismes favorisant leur accès aux vaccins à travers la subvention totale ou partielle du coût du vaccin. Des communications régulières sur le sujet s´avèrent aussi utiles car en dépit des enseignements qu´ils reçoivent sur la maladie, leurs connaissances en la matière demeurent encore limitées.

### Etat des connaissances sur le sujet

- Tous les professionnels doivent être vaccinés contre l´hépatite B;
- En 2010, 47,7% des professionnels de santé avait reçu au moins une dose de vaccin contre l´hépatite B au Burkina Faso;
- La prévalence de l´hépatite B chez les professionnels de santé au Burkina Faso était de 14,5% en 2014.

### Contribution de notre étude à la connaissance

- En 2020, 31,0% des élèves professionnels de santé avait reçu au moins une dose de vaccin contre l´hépatite B;
- Seulement un élève sur trois connait les risques d´exposition à l´hépatite B en milieu de soins;
- Les connaissances des risques d´exposition et des complications sont déterminantes dans la propension des élèves professionnels de santé à être vacciné contre l´hépatite B.
